# The Hospital. Nursing Section

**Published:** 1904-05-14

**Authors:** 


### The Hospital.

**Nursing Section.**

**Contributions for this Section of "The Hospital" should be addressed to the Editor, "The Hospital" Nursing Section, 28 & 29 Southampton Street, Strand, London, W.C.**

No. 920.—Vol. XXXVI. *SATURDAY, MAY 14, 1904.*

### Notes on News from the Nursing World.

### THE KING AND HIS NURSE.

An incident of interest to nurses marked the close of the Royal visit to Ireland. When the King and Queen were leaving Lismore his Majesty sent a telegram requesting that Mr. Charles Haines, the father of Miss Haines, who acted as senior nurse during the King's last dangerous illness, should meet him at Mallow Railway Station. Both the King and Queen shook hands warmly with Mr. Haines and engaged in an animated conversation with him, in the course of which his Majesty alluded most kindly to Miss Haines and expressed a hope that she was very happy in her present work at Osborne. Loud cheers greeted this manifestation of their Majesties' gracious recognition of the services rendered by Miss Haines, of whom Mallow is justly proud. The National Anthem was enthusiastically sung by the people as the Royal train left the station, thus ending an extremely pleasing function.

### ROYALTY AND THE VICTORIAN TRAINED NURSES.

In the autumn of last year Sir George Clarke sent a despatch to the Colonial Secretary recommending the grant of the title "Royal" to the Victorian Trained Nurses' Association, and forwarded for presentation to the Queen an address from the Association in a casket of Victorian wood. The reply of Mr. Lyttelton appears in the March number of the Journal of the Association, from which we learn that on ascertaining that the Governor-General concurred in Sir George Clarke's recommendation the Colonial Secretary submitted the application to the King, who has been graciously pleased to accede thereto. Mr. Lyttelton adds, "The address from the Association has been laid before her Majesty the Queen, who desires me to thank the Association for the sentiments contained therein, as well as for the casket in which it was contained." In consequence of the honour conferred upon the Association by their Majesties, the Council of the Association have decided to provide the members with a new badge. The nurses have therefore been asked to send in designs to the Hon. Secretary, each design to bear the inscription R.V.T.N.A., and to be surmounted by a lion and unicorn on a crown. A sub-committee of ladies will select the six best designs, and the final selection is to be made upon the votes of the nurses themselves.

### POOR-LAW NURSING IN SCOTLAND.

It is satisfactory to learn that the Departmental Committee on Poor-law Medical Relief in Scotland do not in their report, just issued, put forward a proposal similar to that of the English Departmental Committee for the creation of a qualified nurse with a qualification of one year's training. This committee, instead of lowering the standard, would like to raise it; but while convinced that even under existing conditions two years' training should be the minimum, they do not think that the time for insisting upon the extension of the period to three years has yet arrived. They recommend, however, that house committees should be urged to employ, if possible, the better-trained nurses and none who did not hold a two years' certificate. They are also of opinion that one nurse with a three-years' certificate should be employed in each poor-house. With regard to poor-house matrons, they advise that the lady superintendent, or head nurse, should not be allowed to act as matron of the poor-house—who in Scotland has no authority within the hospital—but they affirm that the latter should always be a nurse with three years' training, and also that the matrons of the smaller poor-houses should be fully-trained nurses. Before the President of the Local Government Board comes to a final decision respecting the report of the English Departmental Committee, he would do well to carefully study that of the Scottish Committee, which is distinctly encouraging to all who desire the elevation of Poor-law nursing.

### DEATH OF ANOTHER NURSE FROM PLAGUE.

Only last week we recorded the death of a nurse from plague at Johannesburg. This week we regret to announce that Miss Harriet Winscom, another nurse, has fallen a victim to the disease in India. Miss Winscom, who from the commencement of the epidemic in the Presidency of Bombay, gave all her time, energies, and sympathy to the plague-stricken, herself contracted the disease about Easter, and died after a nine days' struggle, in spite of the skill of Dr. Sarkhad and the care of the sister and nurse in charge. For some years she had been working at the Modi Khana Plague Hospital, which under her careful nursing became a refuge to which the poor stricken natives willingly went and reluctantly left. She had many tales to tell—some were told in our columns, to which Miss Winscom frequently contributed—of their gratitude, and of their simple methods of showing it. If she had lived she would have begun a holiday this month, but death has anticipated for her the rest she so much needed. Her funeral took place on April 12th at Suvre amid many manifestations of regret from all sorts of people.

### A QUESTION FOR THE METROPOLITAN ASYLUMS BOARD.

A serious statement reaches us from a trained nurse who recently left one of the hospitals under the jurisdiction of the Metropolitan Asylums Board. It is to the effect that she "was allowed to go away without any of her clothing being disinfected." In the same hospital, we understand, the in- and out-door clothes of nurses are kept in one room. A similar complaint was made to us a little time since by a nurse who had been in the employ of the Board; but as she seemed to refer to a condition of things which prevailed a couple of years ago, we hoped that it had ceased to exist. It is incredible that the Board can be aware of what can only be characterised as an entire disregard of the elementary precautions which should be taken to prevent the spread of infectious diseases. It must also, we imagine, be absolutely in contradiction to the rules of the institutions under their charge.

### THE MIDWIFE'S AGE.

At the annual meeting of the Rural Midwives' Association, reported in another column, Dr. Boxall said that the question of age was one of great importance. He thinks that a midwife should not be under 21, nor over 45. It seems to us that the limit is too low in both respects. Why should one woman be allowed to become a midwife at 21, when another cannot, even with longer training, become a nurse until she is 26? As to the age not exceeding 45, the work is seldom too arduous for a healthy woman of 55, or even 60, to manage. Many mothers would probably prefer to be attended by a woman of more mature years.

### PRIVATE NURSING IN CHICAGO.

An English-trained nurse who has lately gone out to Chicago, in writing home to forward her year's subscription for The Hospital, says: "I find the nursing papers here are so different that I miss my Hospital more than I can say, and must get you to send it out to me." Most of the nurses with whom she comes in contact seem, she states, to have been trained for two years, and adds that nurses who take up private nursing in Chicago are well paid, but are almost worked to death. When the writer arrived she found it very hard to get to know the doctors, and for the first nine months it was decidedly difficult to make both ends meet, everything, of course, being much more expensive than in England. Her testimony coincides with the advice we have frequently offered to those who wish to try their fortunes abroad. Provided that a nurse is a good nurse, is willing to rough it, and—the latter point is indispensable—has sufficient capital to be able to keep herself if need be for the first year whilst looking for work, there is no reason why she should not succeed in nearly any quarter of the civilised world.

### PROPOSED TRAINING HOME AT SWINDON.

At an influential meeting held in the Town Hall, Swindon, under the auspices of the newly-formed Wiltshire Nursing Association, it was proposed to start a training home for nurses at Swindon. Mrs. Hobhouse, in an interesting speech, said that the average cost of a skilled nurse was £80, and that to ensure the population of Swindon being adequately nursed 10 nurses would require to be engaged. This meant an expenditure of £800 a year. Mrs. Hobhouse, therefore, advocated the foundation of a home which would yield a considerable sum from the fees paid for the training of probationers to be employed in the rural districts. As the Wiltshire Association propose that only highly-trained fully-certificated district nurses shall work in towns with a population of over 5,000, Swindon has everything to gain by the establishment of a training home. The point open to doubt is whether the second and third grades of nurses, which it is intended that the home shall send out into the villages after six months' training, will be equal to their duties.

### ENGLISH NURSES AND THE INTERNATIONAL CONGRESS OF WOMEN.

There will be a considerable representation of English nurses at the meeting of the International Council of Nurses in Berlin next month. We hear from Miss Mollett, matron of the Royal South Hants and Southampton Hospital, that she has already completed the party who are going to the German capital under her auspices, and we are not surprised, for the terms offered are so moderate that they must have been irresistible to some who desired both to attend the Congress and to visit Berlin. It is a considerable advantage to members of the party who do not know German that Miss Mollett speaks the language fluently. The session on trained nursing is to be held on Thursday, June 16.

### AN UNFILLED VACANCY AT ROCHESTER.

There seems to be a scarcity of nurses for infectious diseases. The authorities of St. William's Hospital for Infectious Diseases at Rochester advertised for a charge nurse, and three candidates were selected. But before the final choice could be made two of these had obtained other positions, and the third was not accepted. The practice of nurses applying for more than one post at the same time is evidently increasing, and it indicates that in some branches of work the supply of suitable nurses is not equal to the demand.

### A LENDING LIBRARY OF PROFESSIONAL BOOKS.

A social gathering for nurses was held at Mary-court, Bridgwater, last Friday, when an interesting address on "The Influence of the Mind on the Body, with Reference to Disease," was given by a medical man. The subject of starting a lending library of professional books and magazines for nurses was discussed and favourably received.

### EDINBURGH NURSES' "AT HOME."

The nursing staff of the Edinburgh Fever Hospital have been holding their annual "At Home" in the recreation hall of the Nurses' Home. The large company present included several well-known public men and officials from various other hospitals in the city. Miss Thomas, the matron of the hospital, and Dr. Ker, the medical superintendent, received the guests. All the nurses present were in uniform. Dancing began shortly after seven o'clock and was continued until eleven.

### ASSOCIATION OF ASYLUM WORKERS.

At the annual meeting of the Association of Asylum Workers on Tuesday next, the gold and three silver medals awarded by the Association for long and meritorious nursing service will be presented. We have already given the names of the winners. The meeting takes place at the Medical Society's House, 11 Chandos Street, Cavendish Square, at 4 p.m., and all interested in asylum work and workers are invited to attend.

### DISTRICT NURSING AT LEAMINGTON.

Another instance of the kind of help which may be rendered to a District Nursing Association was afforded at Leamington last week. Under influential auspices a vocal and instrumental concert took place in the town, the programme including the performance of a cantata entitled "May Day," and a number of songs and orchestral selections. At the close of the concert the Rev. E. W. S. Kingdom, Vicar of St. Mary's, described the excellent work which is being done by the three district nurses, and stated that there would be a splendid balance to hand over to the Nursing Association. Loud cheers were given for the nurses.

### A USEFUL SUGGESTION BY A POLITICIAN.

At a tea soirée in aid of the funds of the Bangor Nursing Institute, Mr. R. A. Naylor, the Unionist candidate for the Carnarvon Boroughs, suggested that a valuable addition might be made by associating with it some kind of kitchen, where good and suitable invalid food could be obtained, either gratuitously or for the payment of a very small sum of money. He mentioned that some few years ago a friend of his gave a sum of money to start such a kitchen in a certain town. No one had anything to do with it except the lady herself, the lady superintendent of the nursing institution, and a lady who did the cooking for nothing. Only the other day he saw a letter from the superintendent stating how very much sooner the sick poor were made well when they had a little nourishment in the way of suitable food. The accounts of the same little undertaking showed that about one-fourth of the cost was paid by the patients themselves, but the very poor were supplied with the food gratis. He thought that some one in Bangor might be ready to find the money for such an enterprise, and some one else to do the work, and he believed that it would indirectly lessen the labours of the district nurses.

### THE NEEDS OF MIDDLESBROUGH.

The annual report of the Middlesbrough Nursing Association, which has been forwarded to us for notice, shows that on March 15th last there was a balance of £88 due to the bank. This is all the more to be regretted as there is need in the town for double the number of district nurses, if only funds were forthcoming to supply them. The Association was founded in 1890, the number of cases nursed in that year being 218, and the number of visits paid 4,831. Last year the number of cases nursed was 706, and the number of visits paid 19,138. The nursing staff now consists of the lady superintendent and five nurses, who perform their duties under many difficulties. The support given to the Association is gratefully acknowledged, but until a larger regular income is provided, the position must continue to give cause for anxiety to the managers.

### MALE NURSES AND EDMONTON WORKHOUSE.

It appears that since 1898 no fewer than eight male nurses have been appointed in succession to look after the infirm at Edmonton Workhouse. The committee accordingly recommended that a female nurse should be elected to the last vacancy, which was agreed to. The commencing salary is £22, increasing to £26.

### RECOGNITION OF SERVICES.

At the annual meeting of the subscribers of the Norwood Cottage Hospital on Saturday, Mr. Tritton, M.P., in the chair, Dr. Beaumont moved a vote of thanks to the matron and nursing staff for their "continual kindness and attention to patients." Dr. Gandy seconded the proposition, and said that during the period the present matron had been in office, the hospital had been second to none in its comfort and happiness. He had been at the hospital at all times of the day and night, and the matron was always there, ready to show sympathy and kindness. He might say exactly the same of the nurses. There was a stream of love which seemed to pass to all the suffering. Mr. C. E. Blundell warmly supported the proposition, and maintained that subscribers owe a very great debt of gratitude to their matron. The Chairman said he was sure the resolution commended itself most heartily to them all. On being put to the vote it was carried most cordially.

### A GOOD BEGINNING.

At the first annual meeting of the Stanningley and Farsley District Nursing Association, Mr. T. Rider said that he had enjoyed several opportunities of seeing the work of the nurse during the past twelve months, as he had been in constant attendance on the sick, and he knew of patients who, but for skilled nursing, would have succumbed. The value of the work was attested by other speakers, and the association must be congratulated upon the good start which has been made.

### STOCKPORT HOSPITAL.

In our issue of April 23rd we referred to an inquiry held by a sub-committee of the Stockport Corporation into charges brought against the management of the Stockport Hospital by a nurse who was stated to have been dismissed on the ground of insubordination. This statement was reproduced from a report of the meeting which was published in the columns of a leading and highly trustworthy Manchester paper. Our contemporary has now published another paragraph to the effect that it is informed that its statement that the nurse was dismissed on the ground of insubordination was incorrect, and that her engagement was terminated with the usual notice. Our contemporary has expressed regret at the error, and has apologised to Nurse Griffin for any inconvenience that she may have suffered from it. Having published the statement from the report of our contemporary, we take the earliest opportunity of expressing our regret that it should have been reproduced in our columns and of tendering our apology to Nurse Griffin for the publication of the erroneous statement.

### SHORT ITEMS.

Miss Mary E. Richardson has been provisionally appointed staff nurse and Miss C. F. Gash has resigned her appointment as sister in Queen Alexandra's Imperial Military Nursing Service.—With regard to the appointment of Miss Satchwell as matron of the male department of Stirling District Asylum, Larbert, the vacancy was due to Miss Irvine giving up the position in order to be married.

### The Nursing Outlook.

"From magnanimity, all fear above;

From nobler recompense, above applause,

Which owes to man's short outlook all its charm."

### ANNA STOLBERG.

Sometimes the best way of looking forward is to look back; the past is generally the forecast of the future. And so in the rush of our nursing life, in our pride about the "Twentieth-Century Midwife" and our eagerness about our "profession," we miss perhaps what will ever be the dominate note of nursing, so long as it remains in woman's hands. That note is the joy of self-sacrifice, the glory of humility. It is not the women who clamour and push and struggle for notoriety whose names live after them; it is the great and silent women of whom no newspaper makes mention, whose work is their record, and whose lives are written in the hearts of those who love and follow them.

On September 16th, 1819, was born Anna, eighth child of Count Anton, of Stolberg, in Silesia; the family was very old and very rich. When Anna was in her teens, Count Anton was appointed Chief President of the Rhine Provinces, and the family moved to a house on the borders of the Rhine. Here the Count made the acquaintance of Pastor Fliedner, who unfolded to him his desire to form an order of nursing deaconesses. It was on May 30th, 1836, in the house of Count Anton, at Dusseldorf, that the rules for the "Rheinisch-Westfählischer Diakonessenverein" were drawn up and signed. It was on October 13th of the same year, that Kaiserswerth was opened—Kaiserswerth, where our Florence Nightingale herself went for training. Anna must have heard much about the duties of women towards the sick, and she visited Kaiserswerth constantly. In 1842 Count Anton was appointed Chief Administrator of the Palace at Berlin, and Anna, then 23 years of age, passed into the life of the court; but she found it, according to her own word, a "desert!" But her position brought her certain consolations. When Elizabeth Fry went to Berlin and was received by the Queen, Anna was present, and has left us this picture of our illustrious countrywoman:—"It was a strange sight, at once imposing and attractive, to see this woman of sixty with her plain grey gown, her Quaker bonnet, her glance, soft yet penetrating, seat herself without ceremony between the Queen and the Princess Wilhelmina, addressing them without using any titles the whilst she pleaded the cause of the sick, the poor, the prisoners, and the unhappy of all conditions. She spoke with eloquence, with ease, with reason; and it was evident her heart was warmed with the love of Jesus."

Count Anton called Pastor Fleidner to Court, and in the gardens of Charlottenberg those two and the King Frederic-William paced up and down and formed a scheme for helping the sick of Berlin. In 1847 the King himself solemnly opened the imposing hospital of Bethany and had presented to him the sister-directress Marianne de Rantzaw, and nine other deaconesses from Kaiserswerth, who had undertaken the nursing. There was now an oasis in Anna Stolberg's "desert," and her visits to Bethany were frequent. In June 1853 the splendid carriage of the Stolbergs drove up to the great door of the hospital of Bethany, and the Count and Countess and Anna descended and passed into the chamber in which the sister-directress lay invalided on her bed. "Venerable Sister," said Count Anton, "we bring you our daughter Anna. God has drawn her towards the serving of Him by serving the sick in this hospital. We, her parents, implore the blessing of God on her work."

"Amen!" said the invalid, kissing the girl kneeling by her bed, "your Anna shall be my beloved daughter." With trembling hands she covered Anna's rich hair with the plain white bonnet of the novice and Countess Anna passed from the world to the ward. Sister Anna served well and quietly, and when Mother Marianne de Rantzaw died, it was Mother Anna de Stolberg who was chosen her successor. There were now 300 beds at Bethany and about 150 sisters and novices, the work was heavy. In 1856 Mother Anna visited all the principal hospitals and institutions of Germany, both Catholic and Protestant, in order to profit by the experiences of others, and she ended up by visiting Kaiserswerth once more. In 1864 war broke out, and the Knights of St. John organised the ambulance; Count Everard de Stolberg, a brother of Anna, was at the head of this order, and he sent word, "To our help, Anna! we have need of thee and thy deaconess." At once Anna and some of her helpers set out and organised an ambulance at Altona; Mother Anna was the first deaconess who ever served on the field of battle. It is impossible to follow all her work; enough that when peace was declared two years later, and the King wanted to present medals to Anna and her sister-nurses, the Directress of Bethany refused all worldly honours. The following year in Eastern Russia there was a terrible outbreak of typhus, and once more the cry came from the Knights of St. John, "To our aid, Mother!" Anna started at once, it was even more difficult than organising in war, and under the privations and trials Anna's health broke down, and she contracted the fever and died. The pastor who preached her funeral oration said: "The principal trait of her character was the desire to serve."

### The Modern Nursing of Consumption.

By Dr. Jane H. Walker, Physician to the New Hospital for Women, London, and Medical Superintendant of the East Anglian Sanatorium, Nayland, Suffolk.

### LECTURE V.

### Nursing in Sanatoria for the Poor.

At the risk of repetition, attention must again be directed to the fact that consumption is not an ordinary acute illness short in its duration; it is a long and tedious illness, requiring at least six months for due treatment. It can easily be imagined that to take working men or women away from work, patients whose interests are, to say the least of it, very limited, who are used to waiting on themselves, to living on scanty diet, or at any rate on plain food, and to place such patients in a luxurious home replete with every comfort, where they live like fighting cocks, and are waited on hand and foot, and where they must stay for a considerable time, is demoralising in the extreme, and renders them practically unfit to follow their former occupations when they are well enough to return to them. This being so, the danger must be avoided by arranging sanatoria for poor consumptives on the simplest possible lines. The sanatorium must be furnished as sparsely as can be, the food must be as plain as is consistent with the due proportion of constituents required for a dietary for consumptives, and whenever the patients are able, they must give what assistance is required in the house and garden. All these points make the arrangements in a sanatorium for the consumptive poor very different from those found to work best in an establishment for educated patients. Fewer nurses will be required, and perhaps these should be of rather a different kind. In a sanatorium of, say, eleven beds a nurse who is working matron and housekeeper, with a young probationer under her, and a cook-housemaid, is all the staff required. All but the absolute scrubbing of the house can generally be done by the patients. Great tact and patience is necessary to deal satisfactorily with this part of the work. We all know that it is far easier to do things ourselves than to make others do them, and far more is this the case when the persons we wish to make work are, firstly, not feeling very robust, and secondly, are probably resenting our efforts with all their power. Patients are specially apt to do this where they pay, and still more so where someone else is paying for them. Yet it ought to be done, if the last state of the patients is not to be worse than the first.

Of the household work, dusting with a wet duster, washing up crockery, cleaning boots and knives, laying the table for meals, and clearing it again, and most of the necessary mending and sewing, can be done by the men and women patients. Such help as they can give should be required of them. Equally, whatever garden work can be done by men patients should be enforced. It must be borne in mind that, provoking as well-to-do consumptives are in grumbling over their meals, they are not to be compared with poor patients. This must be faced, and reckoned with by every nurse working in a sanatorium for the consumptive poor.

The food should be as far as possible of such a kind that it is within the means of the patients to procure for themselves when they leave. Suet pudding, haricot beans, lentils, bacon, peas, peaspudding, dripping and so forth, are articles of very high dietetic value, and can be obtained at comparatively cheap rates. A great deal of tact will be needed to make the average person of the artisan class think he is getting his money's worth with such a diet. His one idea of good food seems to consist of anything cooked in a frying-pan, such as fried rumpsteak, but that idiosyncrasy must be prepared for. Statistics as to results obtained in sanatoria for poor consumptives are highly encouraging, being little, if at all, inferior to those in the better-class establishments. If the money for the building and furnishing be found, an institution for twenty to thirty patients, paying 16s. to a pound a week, can be made to pay expenses.

The effects of sanatorium treatment are generally seen very rapidly. The most striking fact about the majority of sanatoria is that the patients look extremely robust, not to say bucolic. They have sunburnt or wind-tanned faces, they are generally well nourished, and there is a remarkable absence about the building of any sound of coughing. Of course there may be bad and even dying cases (for patients are not infrequently sent by their doctors in an absolutely hopeless condition, as if the open air were some miraculous agency, to be turned to when the patient is practically dying), but such patients are in bed; and the general atmosphere of any well-regulated sanatorium is one of cheerfulness and health.

The gain in weight under the treatment is often astonishing, especially at first. One patient we have known gained 12 lbs. in two weeks. As a rule, a steady gain of $1\frac{1}{2}$ to 2 lbs. weekly is to be preferred to such sudden accessions of weight.

### Nursing in Foreign Sanatoria.

This may be described as like the snakes in Iceland. Nurses, as we understand the term, do not exist. The attendants on the patients are servants only, and they have no training or nursing experience whatever. This at least is so at all the sanatoria with which we are acquainted, either by personal observation or by repute. They are very good and kind and do their ignorant best, and are very timid and anxious poor things when they come into contact with the graver and more serious aspects of the disease. When the patient is up and well they clean his room and make his bed; when he is ill they, in addition, bring his food and wait on him more or less, making his bed or not as he wishes. When he is too ill to have any visitors one of them, with the exception, of course, of the doctor, is his only comfort. The servant often does not know his language, and he often does not know more of hers than suffices to ask for the various necessaries. One advantage which English sanatoria, not imitating slavishly everything German, have over foreign sanatoria, is found in the much greater comfort existing in the nursing department.

### To Nurses.

We invite contributions from any of our readers, and shall be glad to pay for "Notes on News from the Nursing World," or for articles describing nursing experiences at home or abroad dealing with any nursing question from an original point of view according to length. The minimum payment is 5s. Contributions on topical subjects are specially welcome. Notices of appointments, letters, entertainments, presentations, and deaths are not paid for, but we are always glad to receive them. All rejected manuscripts are returned in due course, and all payments for manuscripts used are made as early as possible after the beginning of each quarter.

### Curative Treatment in Cases of Bed-sores.

### EXAMINATION QUESTIONS FOR NURSES.

THE question was as follows:—"State what curative treatment you would pursue in cases of slight and also of serious bed-sores."

### FIRST PRIZE.

The first thing to do in any case of bed-sore, whether serious or slight, is to remove pressure from the part and a water-bed is the best means of doing this. If the case is likely to be a lasting one, as myelitis or fractured spine, it is much better to have a full-sized water-bed as other parts of the patient's skin are likely to get sore as well as his back.

It is important that a helpless patient's skin should be examined all over when he is being washed, as bed-sores are not confined alone to the back and heels, but any part of a patient's body is liable to get sore where there is pressure, such as the elbows, shoulders, and behind the ears.

Sometimes where a patient is very emaciated bed-sores form on the iliac prominence, caused by pressure of bone from within.

The main points in the treatment of bed-sores are to attend to the general state of the patient's health, as the better he is nourished the quicker a bed-sore will heal and also to prevent constipation. The site of the bed-sore should be thoroughly cleaned with a mild antiseptic lotion and, if slight, it should be covered with a zinc dressing and a bandage.

If the bed-sore is severe and there is a slough it must be fomented every four hours to get out the slough, but it must be borne in mind that the vitality of the tissues is much lowered, and therefore fomentations must not be applied too hot.

When the sore is clean balsam of Peru may be used on a piece of lint, cut the exact size of the wound, to stimulate and assist the healing process. This dressing should be changed at least twice a day.

If the wound does not get on as fast as it might a change of dressing is often beneficial and a solution of sod. chlor., about 1 in 24, might be tried. A piece of lint, cut the exact size of the sore and soaked in the solution with a piece of jaconet just overlapping the lint, and covered with wool and a bandage. Care must be taken to prevent the lint overlapping the edges of the sore, as it would tend to soften the edges and cause them to break down. The patient must be kept as dry as possible from the effects of urine, sweat, etc., and should be changed whenever there is any necessity. All crumbs should be removed from the sheet and the sheet pulled perfectly straight. "AUBREY."

### SECOND PRIZE.

Bed-sores begin as redness in cases which are confined to bed. In cases of slight bed-sores, *e.g.* where the skin is just broken, I should first of all attend to bed and bed-making. If possible, I should get a water-pillow, and put this with a sheet covering it next to the patient. This I have found more beneficial to the patient than when a mackintosh is put next over water-bed, as water-beds naturally sink a little when a patient is on it, and this tends to ruck the mackintosh, causing discomfort and irritation. Failing to secure a water-pillow, I should make rings of tow covered with old linen so as to relieve the threatened spots. Having got air-cushion, ring-pads, or water-pillow I should then give special attention to bed-making. First, I should avoid lumpiness, wrinkles, and crumbs. Secondly, I should regularly and thoroughly cleanse the parts exposed to pressure by washing with soap and water, using my hand to wash where the skin is broken. I should next try to educate the surrounding skin to withstand pressure by gently rubbing—to stimulate circulation—the unbroken skin with spirits, as methylated or brandy, dusting with fuller's earth, or starch, and zinc powder. To the broken skin I should apply a healing ointment. I have found vaseline mixed with castor oil to a consistency of cream, very beneficial in cases that have not yielded to boric or zinc ointment; fasten this where possible with a bandage, as strapping stuck to delicate skin, which we nearly always get with bed-sores, often leads to a disastrous result. I should change position of patient very frequently. Bed-sores which involve the soft tissues—*e.g.* ulceration and suppuration of the soft tissues—should first be got clean by fomentations. As soon as the slough has come away and the wound is quite clean, I should wash with a stimulating lotion, as carbolic, 1 in 80, and apply a dressing of rubra lotion in a few days. This may be followed with a healing ointment, such as zinc or boric, or whatever kind of ointment the doctor prefers, as I should always report a bed-sore to the doctor. For the unavoidable bed-sores, which I am glad to say I have seldom met, which we find with patients suffering from nervous diseases, the doctor usually likes to prescribe his own mode of treatment. If the bed-sores are very extensive, after getting them clean, iodoform gauze makes a very good dressing, and it has the advantage of rubra lotion, because it is a good antiseptic. It is well to use antiseptics as far as possible when treating bed-sores.

"MOLLIE."

### THE PRIZE WINNERS.

The winner of the first prize is "Aubrey," and he is successful because he considers the question in all aspects and evidently after close and intelligent observation of numerous cases. He mentions one important point generally overlooked by nurses—namely, that if one dressing does not work some improvement, it is advisable, after a trial of a few days, to substitute something else which may suit that particular patient better. It is a great error, simply because a remedy is good in itself, to expect a miracle worked and a cure effected where manifestly the means employed are unsuitable.

"Mollie," the winner of the second prize, sends a good paper, also witnessing to much practical insight. It is quite comforting to hear of castor oil and vaseline, as a variety to the everlasting boracic powder and zinc ointment.

She notes, too, the evil results of the use of strapping.

It is far better to apply dressings every hour to a bed-sore than to risk what is likely to follow the use of strapping on a skin already in a more or less unhealthy and unnatural condition.

### PAPERS ARE ON THE WHOLE GOOD.

A large number of excellent answers are to hand this month and show considerable improvement on those last sent in on the same subject; but it is vexing to see the repetition of the same errors in many cases, such for instance as advocating collodion painted over a commencing sore!

Consider for one moment the impossibility of applying any friction to the surrounding parts, to say nothing of the fact that it would be also out of the question to examine the condition of the sore itself. Strapping, too, is a most dangerous agent—at all times very unpleasant in its action; when placed on an unhealthy back (probably extremely tender from perspiration), the results would be disastrous.

### HONOURABLE MENTION.

This is gained by six nurses. "Belinda," who would have gained a prize, but for clinging to strapping. She very wisely notes the fatal effect of mingling ointment and powder as remedial agents, the effect of such communion being the formation of greasy lumps. "Hopeful," "Trix," "A Climber," "Nil Desperandum," and "Theo."

### NEW RULE.

Take notice of the new rule which prevents any one nurse receiving more than three prizes in the current year.

### QUESTION FOR MAY.

If called upon suddenly—say, with 12 hours' notice—to prepare for an operation for appendicitis in a private house where means are limited, how would you proceed both with regard to the patient and the available rooms? N.B.—You are not asked to describe dressings; they would be brought by the surgeon.
THE EXAMINER.

RULES.

The competition is open to all. Answers must not exceed 500 words, and must be written on one side of the paper only. The pseudonym, as well as the proper name and address, must be written on the *same* paper, and *not on a separate sheet.* Papers may be sent in for fifteen days only from the day of the publication of the question. All illustrations strictly prohibited. Failure to comply with these rules will disqualify the candidate for competition. Prizes will be awarded for the two best answers. Papers to be sent to "The Editor," with "Examination" written on the left-hand corner of the envelope.

In addition to two prizes honourable mention cards will be awarded to those who have sent in exceptionally good papers.

N.B.—The decision of the examiner is final, and no correspondence on the subject can be entertained.

NEW RULE.

Any competitor having gained three prizes within the current year shall be disqualified from taking another until 12 months shall have expired since the first prize was gained.

### Midwifery: as Practised by Native Midwives in Fiji.

By the Hon. B. GLANVILL CORNEY, Chief Medical Officer of the Colony.

(*Concluded from page* 69.)

An occasional circumstance which the midwives seem to misunderstand, and deal wrongly with, is the non-delivery of portions of the membranes. They lay particular stress on the impropriety of removing such fragments—*ai kube-kube*, as they call them, lit., "the cleavings"—even when they have been in part extruded spontaneously and can therefore be seen and grasped. In such an event, on the contrary, they take particular pains to secure them by tying them down *in loco* under a bandage of bark cloth, trusting the sequel to nature. But they admit that women to whom this happens usually remain feverish and ill for some time afterwards, and they evidently consider the situation a precarious one. In fact, this and other features in the masterly inactivity which characterises the practice of the native midwife in a general way may be regarded as the outcome of experience in bygone ages, depending on the unclean condition of the midwife's hands and person—though she herself does not know it, having no idea of asepticity in her benighted mind to guide her actions.

A herbal potion called *wai ni lutu vata* (nostrum for con-descent) is sometimes given during the later months of pregnancy, with the object of favouring an easy labour, and the descent of the placenta together with the passage of the child, or at the right moment immediately afterwards. After the conclusion of the third stage some midwives in the highlands of Na Viti Levu introduce the finger tips into the uterine neck and clear out the clots; others raise the mother to a sitting posture to encourage their discharge from the fundus of the vagina by gravitation—a preferable practice.

In many of the hill districts the mother leaves her hut as early as the day after delivery, and goes about her domestic duties. In other districts they do so somewhat later, generally about the fifth day. But at Bau and among the chiefs and better classes in many localities on the coast a woman not unusually lies on her mat for a much longer time than that, and stays within doors for a whole month. It was formerly the custom, when concubines and slaves were a regular institution, for a mother of any family rank to lie about indoors for 100 days, her affairs being attended to by her domestics and her husband's following. This period was called the *bogi drau*—"hundred nights."

The manner in which Fijian mothers discharge their obligations to their children during the early months of infancy leaves much to be desired. The two great essentials for the survival of the infant are a proper diet and cleanliness—neither of which can be said to be satisfactorily assured. In former times it was the invariable practice to anoint both mother's and child's bodies with oil, and turmeric obtained from the plant which grows freely over the hills and waste places of the islands. The custom is still extensively followed in Polynesia, both in and out of Fiji, as well as in some parts of Asia; but though natives cling to the use of oil, the habit of besmearing the body with turmeric has to some considerable extent been relinquished, owing, it seems, to discredit being thrown upon it by native missionary zealots, who regard it as a relic of the old heathen cult. It is difficult to understand why they should draw the line at turmeric while they by no means disapprove of oil; nor has it been possible to elicit any precise mythological or superstitious reason for the first employment of turmeric in this fashion. The natives say that it is simply used as a cosmetic, and that it promotes a feeling of warmth, comfort and smoothness in the skin; but it certainly was used as an unguent and a colouring agent in the course of heathen rites connected with certain mysteries and debaucheries which used to be permitted to take place at the season of the New Year, the Fijian Yuletide; and the turmeric plant itself held a prominent place in the legend relating to their origin, wherein it seems to be typical of Light, as opposed to the candle-nut tree, from whose fruit lampblack is made, and which was the symbol of Darkness. But, whether Pagan or otherwise in their origin, many of these ancient customs are good in themselves, even though they have in past times been surrounded by objectionable doctrinal colouring; and they may undoubtedly take their origin from material and sound scientific bases.

Such appears, indeed, to be the case with turmeric inunction: the plant belongs to the *Zinziberaceæ*, or ginger family, and affords a mildly stimulating, aromatic, and carminative condiment. Applied externally by means of a lubricant with gentle friction, its effects are feebly antiseptic, cleansing, and soothing, and as such it is healthful and beneficial to the mother and protective to the infant. It has a further use, due to its bright yellow colour, which readily attracts the eye and acts as a signal to the intending visitor or casual passer-by (much as our old-fashioned door-knocker does when tied up) to apprise him that the house is not conveniently accessible for the time being, in consequence of its containing a lying-in patient.

Thus the trained midwifery nurse will gather that her swarthy prototype in far-off Polynesia has little or no knowledge of the theory and mechanism of labour—that she is evilly lacking in antiseptic instinct, and that the situation is saved more by her reluctance to interfere than by any dexterity she may possess or believe she possesses. It is like so many other human affairs, especially those in which man, or woman, presumes to guide the workings of Nature. A little knowledge is a dangerous thing; and in midwifery, if judicious assistance is golden, interference is assuredly of the basest metal unless, indeed, it be applied with the maximum of theoretical knowledge, as well as with all the manual skill to which that knowledge conduces.

To ameliorate the condition of the native women in Fiji, in this as in other directions, the Colonial Government has many well-conceived measures in hand, though the attachment of the native mind to habit and ancient usages renders some of them extremely difficult of execution. But to enter into a description of these would be beyond the province of this paper.

### Rural Midwives' Association.

The first annual meeting of the Rural Midwives' Association was held on Thursday last week at 3 Grosvenor Place, by permission of Lady Esther Smith. Among those present were Lady Esther Smith, Lady Camoys, Lady Balfour of Burleigh, Lady Lucy Hicks-Beach, Viscountess Galway, Lady Emily Digby, Lady Priestley, Lady Elizabeth Byng, Lady Duckworth, Lady Goldney, Lady Mather Jackson, the Dowager Lady Maryon Wilson, Mrs. Hobhouse, Mrs. Helme, and others.

The chair was taken by Sir Michael Foster, M.P., who, in proposing the election of the executive committee, said they had reason to be satisfied with the progress which they had made during the past year in face of many difficulties. One was that the Association, by taking a practical, common-sense view of the situation lost much of the enthusiasm which attended more extravagant ideas. They also ran some danger of being misunderstood. It had been said in some quarters that this Association would hinder the true development of trained midwives, but he entirely denied the charge. It had also been stated that their efforts would tend to put ignorant persons in a position for which they were not fitted by nature, but this again he repudiated, and instanced the care which the Association exercised in the selection of these women as a sufficient answer to this accusation. Yet another charge was that their local committees were in danger of conflict with the local authorities, but he desired to point out that these voluntary committees were instituted more to aid the midwife than to control her. In conclusion, he stated that they had no reason to be discouraged with the results of their first year's work.

Sir John Batty Tuke, M.P., in seconding the motion, stated that he had the deepest sympathy with the objects of the Association, and with the way in which they were carried out. It was the custom in Scotland among large numbers of medical men, both in rural and urban districts, to have on their lists three or four women whom they allocated to various districts as midwives, having been trained either by themselves or in some institution. One essential for all these women was absolute cleanliness, and upon that point he could not lay too much stress; however well a woman might pass an examination, unless she was thoroughly clean she was worse than useless.

Mrs. Heywood Johnstone then read the report which stated that the Association had already sent up 23 women to training institutions. Of these 14 had gone up for the L.O.S. examination, nine of whom were successful, one took the Hackney certificate (recognised by the Central Midwives Board) and four who had failed were working as monthly nurses until May, when, after further teaching, they would try again. Ten midwives and four monthly nurses were therefore at work. Of the remaining nine, one failed in health just before her examination, one had been advised to train as a monthly nurse only, and the others were going up in May or later. Most satisfactory accounts had been received of the trained women now at work. Three candidates were now waiting for vacancies. If subscriptions and donations were maintained the Association hoped to be able to make grants to very poor villages towards training and starting a midwife. A statement of accounts showed a balance in hand of £156, the proceeds of a matinée.

Mrs. Heywood Johnstone went on to say that the gloomy prophecies with which the Midwives Act had been received in some quarters had not been fulfilled. Quite 4,000 women had been registered and were willing to submit to surveillance, still this was nothing in comparison to the needs that had to be met. The Association, however, were grateful for the past and hopeful for the future. They trusted that their members would take a strong practical interest in the work, and manifest it not only by their subscriptions, but also by giving advice and suggestions from their different localities. There was great difference of opinion as to the advisability of the midwives attending general cases. The Association wished to keep on broad lines, and strove as much as possible to leave all questions of detail to the various localities to decide for themselves. Their only proviso was that they refused to supply midwives unless the local committee made some rule to prevent the midwives attending cases that would be dangerous. The Association might find it necessary to raise the cost of training. The committee earnestly appealed for help to enable them to extend the work into out-of-the-way districts that could not afford to start alone.

Dr. R. Boxall said that there were several points to be considered in the selection of candidates, one of which was age. A midwife should not be under 21 nor over 45. She should also be a woman of good character and robust health, be gifted with a certain amount of good sense and tact, and have had a fair education. The local practitioner was the proper person to give advice as to the selection of candidates for midwifery.

Mr. Heywood Johnstone, M.P., also spoke in support of the work of the Association, and votes of thanks were adopted to Sir Michael Foster and Lady Esther Smith.

### The Nurses' Co-operation: Presentation to Miss Roberts.

On Friday afternoon last week Miss E. M. Roberts, the retiring lady superintendent of the Nurses' Co-operation, was the recipient of a handsome present from the nurses. The presentation, which took place at the Howard de Walden Club, was attended by a large number of members of the Co-operation, who were received by the home sister, Miss L. R. Baker, and the secretary, Miss H. F. Gethen. After tea in the refreshment room, a concert took place, consisting of piano solos by Miss Marshall, songs by Mrs. Martin and by Miss Florence Muir, the latter being accompanied by Dr. Arthur Haydon, who played a violin obligato, as well as several solos on the violin.

The presentation was made, in the name of the nurses, by Miss Hallet-Baker, the nurses' representative, and Miss Bryant, one of the resident nurses, each of whom made a neat little speech, regretting Miss Roberts' retirement, and thanking her warmly for her kindly tact and good management, which had contributed in so large a measure to the harmonious working of the co-operation. Miss Hallet-Baker added that the nurses were glad to know that their late matron still remained a member of the co-operation; they hoped that she would enjoy the leisure time before her, and that the piano, which they asked her to accept with their love, would give her many pleasant hours.

After the applause which greeted these remarks had subsided, Miss Roberts, who was holding a bouquet, presented to her on her entry, of red and white roses, lilies of the valley, and smilax, tied with broad red and white ribbons, said this was the most embarrassing moment of her life. She did not know what she had done to deserve so much honour, and could only thank the nurses most warmly for their gift. The piano would be a great source of joy to her and to her friends, and she should prize it not only for its intrinsic worth, though that she knew was great, but also because it was the spontaneous token of affection on the part of the donors. Her services, she continued, had been alluded to in terms far too glowing

("No, no," from the nurses). She had been very happy in her work, and regretted extremely giving it up. Looking upon the vast crowd before her, she could not help thinking what a mighty force the co-operation was, and she could not take leave of the nurses without saying that its success depended upon each individual member of it. The office staff might do their best to fit the nurse to the case, and *vice versâ*, but it was for the members to keep the standard high, and to see that the body to which they belonged maintained the position it had won, in the face of great and increasing competition. It had begun with success, and she looked forward confidently to that success being maintained. Her debt of gratitude extended to the committee, between whom and herself the most cordial relations existed, and whose help she had highly valued during her term of office. Alluding again to the presentation, Miss Roberts declared that she had not thanked the nurses at all adequately. To her remark that the gift was far too good the donors protested, laughingly, and Miss Roberts concluded by saying that she hoped the nurses would visit her in her new home at Wimbledon, though not, she added, in such large numbers as she saw before her in the club-room.

Miss Winning then proposed a vote of thanks to Miss Hallet-Baker for the very able speech in which she had made the presentation. This was seconded by Miss Spinks, and carried unanimously. Other votes of thanks were to Miss Bryant, to the home sister, and to the assistant secretary (Mrs. Crowe). The musical part of the programme was then proceeded with, including recitations by Mr. Herbert Crouch and a piano solo by Miss Cook.

The piano presented to Miss Roberts was inspected at Messrs. Edwin D. Lloyd's, 83 Mortimer Street, both before and after the ceremony, by a large number of nurses. It is a Steinweg Nachf, in rosewood. On the upright part above the keyboard is the following inscription on a small brass plate:—"Presented to Miss Roberts by her friends on the staff of the Nurses' Co-operation, as a token of their warm appreciation of her devotion to their interests. May 1904."

### Everybody's Opinion.

### THE COLONIAL NURSING ASSOCIATION.

"A NURSE IN THE FAR EAST," on behalf of herself and three other nurses, writes: I wish to advise all nurses who think of joining the Colonial Nursing Association to be quite certain before doing so that their contract is perfectly legal, and also to insist upon seeing a copy of the rules of the local, as well as of the central, committee.

### STATE REGISTRATION.

DR. D. G. THOMSON, of Norfolk County Asylum, writes: All the arguments used against the State registration of nurses apply, and were applied with equal force, to the registration of medical practitioners when it was first mooted 50 years ago, and yet would any of us medical men like, either for their own sake or for that of the public, to return to the chaotic conditions of pre-registration days? Let the opponents of the State registration of nurses ponder over this. I know what it is to be a patient as well as a doctor, and as such prize technical ability above every other personal or social qualification.

### PRINCESS MARY VILLAGE HOMES.

"CHARGE NURSE" writes: I should like to hear the opinion of my fellow-nurses on this question. I was appointed last year at the Princess Mary Village Homes, Addlestone, as nurse-in-charge of the infirmary and to have entire charge of all the children (in regard to health) when the lady superintendent was off duty—holidays, etc. I am fully-trained and hold a three-years' certificate from a large training-school. Last January a new lady superintendent was appointed; also, the lady clerk was appointed assistant superintendent and she is not a trained nurse. I have just been informed by the superintendent that I must resign or work under the assistant. The cause of this is because I sent for the doctor of the homes to see a child I had taken from a cottage of 15 children into the infirmary with a temperature of 103° and a throat looking suspicious. The doctor came and ordered treatment and told me to isolate her. I have sent for the doctor before if a case has arisen and have never been reprimanded for it; now I am told that I must tell the assistant superintendent about the case, etc., as she is in charge of all the homes and the infirmary when the lady superintendent is away.

### MILK STERILISATION.

DR. HUBERT ARMSTRONG, Hon. Assistant Physician of Liverpool Infirmary for Children, writes: I notice that on page 78 of your issue of April 30, in reply to a question *re* sterilisation of milk, you state that if the method given is properly carried out "the milk will keep good for many days." This is doubtless a satisfactory proof of the complete sterilisation attained by the process, but I think a word of warning is not out of place against the practice of so keeping it before use, especially in the case of "a delicate baby." From clinical experience I am firmly convinced that the many unsatisfactory features exhibited by the use of sterilised milk as an infant's diet—the too frequent dyspepsia and anæmia and possibly the (increasing) incidence of scurvy rickets—are in a great measure due to the fact that such milk as ordinarily purveyed is only too often "stale." This disadvantage may be obviated if the sterilisation is done at home, provided that only enough is prepared for the maximum period of twenty-four hours, and preferably half that time. With the assurance that the milk will keep indefinitely there is a great temptation, by sterilising several days' supply at one time, to save trouble to the ultimate detriment of the small consumer.

### Appointments.

DORCHESTER ASYLUM.—Miss E. Williams has been appointed matron to the new house, "Herrison," Dorchester, for private patients. She was trained at Dorchester, and has been in charge of the department for lady patients since 1895.

LIVERPOOL CITY FEVER HOSPITAL, NORTH.—Miss Constance Smith has been appointed charge nurse. She was trained at Mill Road Infirmary, Liverpool.

MEMORIAL COTTAGE HOSPITAL, ST. ANDREWS.—Miss R. C. M. Bird has been appointed nurse-matron. She was trained at St. Thomas's Hospital, London, and has since been sister at the Hospital for Women, Liverpool, sister at the Deaconess Hospital, Edinburgh, and nurse-matron at Axminster Cottage Hospital

NEWPORT (MONMOUTHSHIRE) UNION.—Miss Ethel Mary Arr has been appointed charge nurse. She was trained at Newport Workhouse Infirmary.

QUEEN ALEXANDRA'S IMPERIAL MILITARY NURSING SERVICE FOR INDIA.—Miss L. A. Tompkins and Miss W. M. Aldridge have been appointed sisters. Miss Tompkins was trained at Guy's Hospital, London, and has since been relief sister at the Seamen's Hospital, Greenwich, and sister in the Refugee Camps, South Africa. She holds the L.O.S. certificate. Miss Aldridge was trained at King's College Hospital, London, and also in midwifery at the City of London Lying-in Hospital. She has since been staff nurse at St. George's Hospital, London, sister at the Victoria Hospital for Children, Chelsea, sister at the University College Hospital, London, and sister in the Refugee Camps, South Africa. She holds the L O.S. certificate.

WIRRAL CHILDREN'S HOSPITAL, BIRKENHEAD.—Miss E. Hounsfield has been appointed sister. She was trained at Derbyshire Royal Infirmary.

### Echoes from the Outside World.

### Movements of Royalty.

THE King and Queen returned to London on Thursday of last week, looking exceedingly well, and attended the Royal Italian opera at Covent Garden Theatre the same evening. On Monday his Majesty, with the Duke of Connaught—who has entered upon his duties as Inspector-General of the Forces—visited Aldershot to witness the tactical manœuvres of the First Army Corps, which they watched from Danger Hill, Cove Common. The special idea of the operations was that a retreating army from Reading, which had taken up a strong position at Cæsar Camp, was pursued by a northern army advancing from the direction of Cove and Bramshot. The Cavalry Brigade was ordered to prevent the crossing of the Basingstoke Canal by the latter. Subsequently, from a marquee placed on Long Hill, the King witnessed a brilliant cavalry charge across the Long Valley.

A year ago the Prince and Princess of Wales laid the foundation-stone of the Westminster Workmen's Dwellings; on Monday they paid a visit of inspection to the completed and largely-occupied buildings. Only those whose work lies within the boundaries of the City are allowed to occupy rooms in the dwellings, and rents range from 3s. for a one-room tenement to 12s. for four rooms. These sums include chimney-sweeping and the free use of Venetian blinds, baths, hot-water supplies, and drying ground. Their Royal Highnesses first entered some empty rooms, then crossed the roadway and went into a similar set in full cccupation. Amongst the rooms they visited were those of a cook and carver at Victoria Station, a farrier, a waiter, and a railway speedily putting their hosts at ease. The Princess especially noted the hanging conveniences in the bedrooms, the cup-porter. They showed the keenest interest in all that they saw, boards and the gas-cooking arrangements for summer use in the living rooms, and afterwards went to the bath-rooms and the laundries. Before leaving the Prince congratulated the Mayor and Mr. Tozer on the hopeful beginning of what seems likely to be a useful and successful undertaking.

### The War in the Far East.

AN official report of the landing of Japanese troops at Liau-tung Peninsula was received in London on Friday night. The troops were preceded by a landing party of sailors, who had to wade through the water for a distance of a thousand mètres, but were able to take possession of a range of hills without encountering any resistance, and planted the Japanese flag. The disembarkation of the troops was afterwards proceeded with. The Russians do not appear to have opposed the operation. General Kuroki estimates the total Russian casualties in the battle of May 1 as over 3,000. The Japanese have buried about 1,400 Russian dead and have 503 wounded in their field hospital. General Kuropatkin announces to the Czar the Russian retirement from Feng-hwang-chenn. Admiral Togo reports to the Japanese Government that the third operation for blocking Port Arthur was carried out by the combined fleet on May 3. He considers that the entrance is now effectively closed, at least for battle-ships and cruisers. Admiral Alexeieff has telegraphed to the Czar that railway communication with Port Arthur was restored on Monday night. Energetic measures are being taken to hurry forward reinforcements from Russia to the Far East.

### The Late Sir Henry Stanley.

OF the childhood of Sir Henry Stanley, the distinguished African explorer, who died on Tuesday morning, very little is known, except that he was admitted into the Workhouse of St. Asaph at a very early age under the name of John Rowlands. From the workhouse he went as cabin boy on a sailing vessel to New Orleans. There he was given employment by a man named Henry M. Stanley, who ultimately adopted him. His benefactor died, leaving him with no provision, and he had to sell newspapers in the streets for a living The outbreak of the Civil War gave him a chance, he fought on the Confederate side, and ultimately became a war correspondent, in which capacity he went afterwards with the British Expedition to Magdala. His boldness and daring and his attractive mode of writing, caused the proprietor of the *New York Herald* to commission him to go and find Livingstone. At the end of his successful quest he wrote a book "How I Found Livingstone." He next accompanied Sir Garnet Wolseley to Kumasi, and later returned to the interior of Africa, to take up Livingstone's geographical researches. The narrative of his work is given in "Through the Dark Continent." His third journey was in search of Emin Pasha, and how he fulfilled his mission he narrates in "In Darkest Africa." Upon his return in 1890 he became the hero of the hour. Subsequently he married Miss Dorothy Tennant, and from 1895 to 1900 was a member of the House of Commons.

### A Great Hungarian Novelist.

BY the death of Maurus Jokai at Budapest last week in his 80th year, Hungary has lost her greatest novelist, He belonged to a noble Hungarian family, and was educated for the Bar, but early in life turned his attention to literature and journalism. Jokai was a devoted patriot, and took up arms with Kossuth in 1849. When Constitutional Government was restored to Hungary in 1867 he entered Parliament, and both as a debater in the Diet and as the editor of a leading Hungarian paper played an important part on the political stage. But it is as an author that he is best known. Of his earlier romances "Working Days," "The Golden Age of Transylvania," and "The Turks in Hungary" are the most popular. "A Man of Gold" has been translated into English under the title of "Timar's Two Worlds," and "The Lady with the Two Eyes Like the Sea" won for him Academical honours in 1890. His funeral was the grandest ever seen in Budapest. The hearse was drawn by eight horses, and the members of a literary society walked on either side with blazing torches. Over 600 wreaths were carried in twenty carriages, and hundreds of thousands of mourners lined the streets or took part in the procession. Twenty-four gipsies played three of his favourite airs over his grave.

### Royal Italian Opera.

THE Opera season opened this year earlier than usual, and the Opera Syndicate—who have taken Covent Garden—have, for certain nights, increased the prices charged for seats because, they state, the series of operas by Mozart and by Wagner which they intend to represent are to be given in their entirety, and not "cut," as is usually the case. The orchestra on these special nights is to be conducted by Dr. Richter. The part of Donna Anna was taken by Fraulein Destinn, and she was a complete success. Zerlina was represented by Miss Alice Nielsen. The Don himself was M. Renaud, who is not new to the part, and Leporello by M. Journet. Later in the week "Tannhaüser" was given with only one small omission, and was a remarkably fine performance, the King and Queen being present. The playing of the orchestra was magnificent, and Fraulein Ternina made a singularly saintly and noble Elizabeth, singing the celebrated prayer in a manner not easily forgotten.

### The Whitsuntide Holidays.

THE sailings of the popular passenger steamers *Royal Sovereign* and *Koh-i-Noor*, under the auspices of the New Palace Steamers, Limited, will commence on Saturday, the 21st. They will start, as usual, from the Old Swan Pier, London Bridge, at 8.30 and 9.20 A.M., and will sail to Southend, Margate, and Ramsgate. The fares will also be the same as formerly.

### Notes and Queries.

**FOR REGULATIONS SEE PAGE 12.**

*A Question of Responsibility.*

(43) A nurse from an Institute was sent to nurse a case of infectious disease at a fever hospital, and whilst there she contracted the disease. Is the local authority bound to pay the nurse's fees during the whole time she is at the hospital, or does the authority's liability cease when she becomes a patient?—*A. J.*

It is the usual custom in well-managed institutions to pay a nurse's fees during the whole time she is at the hospital, even though she may be temporarily disabled through contracting illness in the course of her work.

*Soldiers and Sailors' Institute.*

(44) Will you kindly give me the address of the Soldiers and Sailors' Families Nursing Institute?—*N. H.*

Address the Secretary, Soldiers and Sailors' Families Association, 23 Queen Anne's Gate, Westminster, S.W.

*Sick Nurses.*

(45) Will you kindly tell me to whom to apply for information respecting the Sick Nurses' Benefit Fund?—*R. A. D.*

There is sickness insurance in connection with the Royal National Pension Fund for Nurses which also has a Benevolent Fund to assist sick members. We do not know of any sick benefit fund for nurses in general.

*Elastic Stocking. M. A. B.*

(46) 1. Please tell me where I can purchase an elastic stocking. 2. And give the names of some of the Metropolitan Asylums Board fever hospitals.—*L. W.*

1. Elastic stockings may be bought at any of the stores or instrument makers. Ask your local chemist to get you one. 2. The Brook Fever Hospital, Shooter's Hill, Woolwich; the Eastern Fever Hospital, The Grove, Homerton, N.E., etc.

*Short Training.*

(47) Will you let me know at what hospital I can obtain a certificate at the end of three or six months' training?—*B. S. A.*

No certificate in general nursing can be obtained for so short a training. The Maternity Charity and District Nurses' Home, Howard's Road, Plaistow, E., grants a certificate for maternity and district work at the end of short periods of training.

*Hospital Training.*

(48) Will you tell me at which of the following hospitals a probationer would get the best training: North-Eastern Hospital for Children, N.E., the Plaistow Fever Hospital, E., or the Royal Hospital for Children, Glasgow? I want to go into either Bart's. or Guy's later, and I should like to know what training would be most useful to me afterwards.—*M. W.*

Write and ask the matron of either St. Bartholomew's or Guy's from which preliminary school she would prefer to take you.

*Home.*

(49) Can you tell me of a nursing home in the country (bracing) where the Weir-Mitchell treatment is given?—*A. F. W.*

We do not recommend private homes.

*Evening Classes.*

(50) Will you kindly tell me where I can obtain evening instruction for the Apothecaries' Hall examination? I have applied to the colleges mentioned in The Hospital, but find that the classes are held during the day.—*E. B.*

Write to the Birkbeck Literary and Scientific Institution, Bream's Buildings, Chancery Lane, or advertise.

### Handbooks for Nurses.

"Nursing Profession: How and Where to Train." 2s. net.; 2s. 4d. post free.

"Nurses' Dictionary of Medical Terms and Nursing Treatment. By Honnor Morten. 2s. post free.

"A Handbook for Nurses." By J. K. Watson, M.D. 5s. net: 5s. 4d. post free.

"Practical Guide to Surgical Bandaging and Dressings." By Wm. Johnson Smith, F.R.C.S. 2s. post free.

"The Examination of Urine." By J. K. Watson, M.D. 1s. net; 1s. 1d. post free.

"Case-book for Private Nursing." 3d. net; 4½d. post free per copy; or per doz. 3s., post free.

The above works are obtainable of all booksellers, or direct from The Scientific Press, Limited, 28 & 29 Southampton Street, Strand, London, W.C.

### For Reading to the Sick.

"BEHOLD I STAND AT THE DOOR."

Before the door of every heart,
Oppressed with sin and care,
A loving Saviour stands and knocks,
Entreating entrance there.

So many days and nights have passed,
So many years have gone,
And yet He patient lingers there,
And gently knocketh on.

The frost has chilled those bleeding wounds,
That tell us of His Love—
All borne that we might dwell with Him
In happy homes above.

How weary are those tender feet,
How sad that loving face,
How numbed those hands that long to clasp
Us in a fond embrace!

O heart of mine, and canst thou bid
Thy loving Saviour wait
Mid winter's cold and frost and snow
Still knocking at thy gate?

*E. J. B.*

"Oh my Father, Thou layest a heavy cross upon Thy poor weak servant, yet I would humbly bow to Thy will, and say, 'Thy will be done.' The cross is no heavier than I deserve. Nay, what do I not deserve for all my sins and wilfulness? And were the cross heavier than it is, it would be nothing to the Cross which Jesus bore for me. O my Father, strengthen my weakness to bear it. It is well surely to suffer thus, or Thou wouldst not lay it upon me. Only deliver me not into the bitter pains of eternal death. This cannot last long. It will look but like a moment when I look back upon it from eternity."—*Anon.*

If interior troubles keep the soul "low," instead of fretting against them, irritated because we are irritable, cross because we feel unamiable, distracted because our thoughts wander, we would humble ourselves before God, and accept the trial as a corrective, it would turn to blessing most assuredly, and we should go on, it might be in twilight, but certainly not in utter darkness.—*S. Lear.*

It is a great gift though, it must be owned, a rare one, to be able to keep silence as to the various minor troubles and vexations which beset our lives. No doubt it is a great relief to tell these to a sympathising ear; but are we not too shy about telling them to the dear Master Who despises nothing that touches His servants' welfare?—*S. Lear.*

We need the conviction that we are not really alone. "Ye shall be scattered," our Lord says to His Apostles, "every man to his own, and shall leave Me alone." And then He adds, "And yet I am not alone, because the Father is with Me." Isolation is meant to throw us back increasingly on God.—*Randolph.*

O Merciful Redeemer, grant us repentance early in the morning, repentance at the third hour, at the sixth hour, at the ninth hour. O most merciful Saviour, grant us repentance at the eleventh hour, grant us the eleventh hour for repentance. According to Thy mercy saving us, Thou Who hast died our death and paid our penalty.—*Christina Rossetti.*

